# Cycloastragenol Attenuates Osteoclastogenesis and Bone Loss by Targeting RANKL-Induced Nrf2/Keap1/ARE, NF-κB, Calcium, and NFATc1 Pathways

**DOI:** 10.3389/fphar.2021.810322

**Published:** 2022-01-20

**Authors:** Gang Wang, Chao Ma, Kai Chen, Ziyi Wang, Heng Qiu, Delong Chen, Jianbo He, Cheng Zhang, Ding Guo, Boyong Lai, Shuangxiao Zhang, Linfeng Huang, Fan Yang, Jinbo Yuan, Leilei Chen, Wei He, Jiake Xu

**Affiliations:** ^1^ First Affiliated Hospital, Guangzhou University of Chinese Medicine, Guangdong, China; ^2^ School of Biomedical Sciences, The University of WA, Perth, WA, Australia; ^3^ Guangzhou University of Chinese Medicine, Guangdong, China; ^4^ Third Affiliated Hospital, Guangzhou University of Chinese Medicine, Guangdong, China

**Keywords:** cycloastragenol, osteoclast, RANKL, Nfatc1, ROS

## Abstract

Osteoporosis, which typically affects postmenopausal women, is an osteolytic disease due to over-activation of osteoclasts. However, current drugs targeting osteoclast inhibition face various side effects, making natural compounds with great interest as alternative treatment options. Cycloastragenol (CAG) is a triterpenoid with multiple biological activities. Previously, CAG’s activity against aging-related osteoporosis was reported, but the mechanisms of actions for the activities were not understood. This study demonstrated that CAG dose-dependently inhibited osteoclast formation in receptor activator of nuclear factor-κB ligand (RANKL)-stimulated bone marrow macrophage (BMMs). Mechanism studies showed that CAG inhibited NF-κB, calcium, and nuclear factor of activated T cells 1 (NFATc1) pathways. Additionally, CAG also promoted the nuclear factor-erythroid 2-related factor 2 (Nrf2)/Kelch-like ECH-associated protein 1 (Keap1)/anti-oxidative response element (ARE) pathway that scavenges reactive oxygen species (ROS). Furthermore, CAG was also found to prevent bone loss of postmenopausal osteoporosis (PMO) in a preclinical model of ovariectomized (OVX) mice. Collectively, our research confirms that CAG inhibits the formation and function of osteoclasts by regulating RANKL-induced intracellular signaling pathways, which may represent a promising alternative for the therapy of osteoclast-related disease.

## Introduction

Osteoporosis is a systemic disease designated by lowered bone mass and microstructure damage, usually resulting in an increased risk of fractures (No-Authors-Listed, 1993). Compared with men, menopausal women face a higher incidence of osteoporotic fractures (van Staa et al., 2001). The underlying mechanism of PMO is that the bone remodeling becomes a negative balance, among which the osteoclast bone resorption exceeds that of osteoblast bone formation (Garnero et al., 1996). Although mainstream drugs that target inhibiting fracture risks in patients with PMO (Black et al., 1996), their side effects cast uncertainty on the use of these drugs and reduce public acceptance (Jha et al., 2015; Black et al., 2020). Due to their safety and effectiveness on bone-related disease, natural compounds with inhibitory effects on osteoclasts have been viewed as alternatives for PMO (Wang et al., 2017; He et al., 2019), and the related compound screening is now a high priority on the task.

Osteoclasts are motile and multinucleated cells (MNCs) derived from mononuclear/macrophage lineage. The directional commitment of osteoclast precursors relies on the aegis of two master cytokines, namely macrophage colony-stimulating factor (M-CSF) and RANKL (Lacey et al., 1998; Arai et al., 1999). The stimulation of M-CSF causes osteoclast precursor cells to express RANK on their membrane that can respond to RANKL, thereby stimulating intracellular signal cascades. With a binding site specific for the cytoplasmic domain of RANK, TNF receptor-associated factor 6 (TRAF6) plays a pivotal role in the activation of downstream pathways, including ROS, NF-κB, mitogen-activated protein kinases (MAPKs), and NFATc1 (Gohda et al., 2005; Lee et al., 2005; Lamothe et al., 2007; Lee et al., 2016). The calcium (Ca^2+^) mediated calcineurin pathway is highly involved in the nuclei translocation and activation of NFATc1, a master regulator of osteoclastogenesis (Negishi-Koga and Takayanagi, 2009). Noteworthy, calcium also regulates the expression of c-Fos, a significant component of activator protein-1 (AP-1), through a non-calcineurin dependent pathway, which promotes the initial induction and auto-amplification of NFATc1 and thus the expression of osteoclast-specific genes like *Acid phosphatase 5* (*Acp5*), *Matrix metalloprotein 9* (*Mmp9*) (Sundaram et al., 2007), *vacuolar H+ transporting ATPase V0 subunit D2* (*Atp6v0d2*) (Feng et al., 2009), *Cathepsin K* (*Ctsk*) (Pang et al., 2019). Besides, the inhibitory effect of NRF2 on ROS production negatively regulates RANKL-induced formation and activity of osteoclast (Hyeon et al., 2013). Collectively, these pieces of evidence suggest that the study of RANKL-induced pathways is essential for screening anti-osteoclast drugs.

CAG is a triterpenoid saponin derived from *Astragali Radix*, a Traditional Chinese Medicine used for thousands of years in treating various diseases. Known as the only natural telomerase activator, CAG has a large body of biological activities, including but not limited to anti-aging, anti-inflammation, anti-oxidant, anti-bacterial, wound healing, and organ protection (Yu et al., 2018). Interestingly, a recent study showed that CAG protected rats from bone loss caused by D-galactose-induced aging or natural aging (Yu et al., 2020); *In vitro* study proved that there exists a promotion effect of CAG on the osteoblastogenesis of MC3T3-E1 cells (Yu et al., 2020). Recently, CAG was also proved to mitigate the suppression of osteogenic differentiation induced by dexamethasone both in MC3T3-E1 cells and zebrafish larvae (Wu et al., 2021). However, its roles in PMO and RANKL-induced osteoclast activities and intracellular signaling pathways are unknown. In this study, we examine the inhibitory effect and underlying mechanism of CAG on RANKL-induced osteoclastogenesis *in vitro* and its protective role in OVX-induced bone loss *in vivo*.

## Materials and Methods

### Materials and Reagents

CAG ordered from Herbpurify CO, LTD. (Chengdu, China) was dissolved in phosphate-buffered saline (PBS) to a storage concentration of 100 mM and further diluted with PBS to different concentrations. Fetal bovine serum (FBS), alpha-modified minimal essential medium (α-MEM), penicillin/streptomycin (P/S) were produced by Thermo Fisher Scientific (TFS) (Carlsbad, CA). M-CSF and RANKL for *in vitro* osteoclast culture were used following previous protocols (Ladner et al., 1988; Xu et al., 2000). Primary antibodies targeting β-actin (Cat# J0914), NFATc1 (Cat# G3014), and IκB-α (Cat# L2010) were purchased from Santa Cruz (San Jose, CA), and the antibody of c-Fos (Cat# 2250S) was from Cell Signaling Technology (Beverly, MA). Secondary antibodies were delivered from BD Pharmingen (San Diego, CA).

### Cell Isolation and Cytotoxic Assay

BMMs from the C57BL/6J mice femur were seeded in culture flasks with α-MEM containing 1% P/S, 10% FBS, and 50 ng/ml M-CSF, passaged cells of 1–3 generations were used in following experiments. BMMs were seeded on a 96-well plate with a cell density of 5×10^3^ cells/well for cytotoxic evaluation. After overnight adhesion, cells were treated by the stated doses of CAG (0, 1, 2.5, 5, 7.5, 10 μM) for 48 h. Under the dark condition, MTS assay buffer (Promega, Sydney, Australia) was dropped to each well for incubation of 2 h. The absorbance value of each well was measured by a plate reader (BMG, Ortenberg, Germany) at a wavelength of 490 nm.

### Osteoclast Differentiation Assay

BMMs culture was conducted on a 96-well plate with 5×10^3^ cells in each well. CAG from 1 to 10 μM was applied to treat RANKL-stimulated BMMs for effect potency screening for 6 days. Both CAG and RANKL were re-added every 48 h. For the evaluation of effect on different stages of osteoclastogenesis, both CAG (10 μM) and RANKL (50 ng/ml) were added to the culture medium of BMMs at the corresponding stage of osteoclastogenesis, which were early (day 1–3), middle (day 3–5), late (day 5–6) and whole stage (day 1–6), respectively. On the sixth day, MNCs (>3 nuclei) of positive tartrate-resistant acid phosphatase (TRAcP) staining under a light microscope were scored as osteoclasts on ImageJ (NIH, Bethesda, MD).

### F-Actin Ring Formation Assay

RANKL-induced differentiation of BMMs was carried out on a 96-well plate for 6 days, and CAG (5, 10 μM) was added together with RANKL. On the sixth day, cells were fixed by 4% paraformaldehyde when mature osteoclasts were visualized. These cells then underwent routine membrane permeabilization by 0.1% TritonX-100, blocking by 3% bovine serum albumin, F-actin staining by rhodamine-phalloidin (TFS, Eugene, OR), and counterstaining by 4′,6-diamidino-2-phenylindole (DAPI). After triple washing of PBS, cells were immediately pictured using a confocal microscope (Nikon, Tokyo, Japan). Measurement of the area surrounded by the F-actin ring and the count of the nuclei number of each osteoclast was accomplished using ImageJ.

### Hydroxyapatite Pit-Forming Assay

With 1×10^5^ cells in each well, BMMs were induced to differentiate by RANKL on a collagen-coated 6-well plate. When osteoclast-like cells were found, cells were trypsin-digested, centrifuged, and resuspended. With 5×10^3^ cells per well, osteoclast-like cells were transplanted to a 96-well plate with hydroxyapatite coating. RANKL was added to cells from the second day with the addition of CAG (5, 10 μM) until mature osteoclasts were identified in the CAG untreated cells. Afterward, half of the wells in each group were washed alternately with sodium hypochlorite and ddH_2_O to expose the eroded area fully. TRAcP staining was conducted on the remaining wells. Measurement of the pit area eroded by osteoclast was completed by using ImageJ.

### Real-Time qPCR Assay

BMMs were seeded on 6-well plates with a cell number of 1×10^4^ per well. During the next 6 days, CAG of 5 and 10 μM was applied to RANKL-stimulated BMMs. Both CAG and RANKL in the culture medium were replaced every 48 h. On the sixth day, mRNA extracted from cell lysates was reverse transcribed as cDNA. A cocktail containing cDNA diluents, SYBR Green MasterMix, and paired primers (Table 1) of target genes were loaded to a 384-well microplate. PCR amplification was conducted on a ViiA 7 system (Applied Biosystems, Warrington, United Kingdom) under the following settings: 94°C for 10 min, 95°C for 40 cycles for 15 s, 60°C for 60 s. The relative mRNA level of target genes was obtained using the 2-ΔΔCT method and then expressed as fold changes relative to the housekeeping gene.

**TABLE 1:** Primers for qRT-PCR assay.

Genes	Forward primer	Reverse primer
*C-fos*	5′-GCG​AGC​AAC​TGA​GAA​GAC-3′	5′-TTG​AAA​CCC​GAG​AAC​ATC-3′
*Nfatc1*	5′-CA ACG​CCC​TGA​CCA​CCG​ATA​G-3′	5′-GGC​TGC​CTT​CCG​TCT​CAT​AGT-3′
*Acp5*	5′-TGT​GGC​CAT​CTT​TAT​GCT-3′	5′-GTC​ATT​TCT​TTG​GGG​CTT-3′
*Ctsk*	5′-GGG​AGA​AAA​ACC​TGA​AGC-3′	5′-ATT​CTG​GGG​ACT​CAG​AGC-3′
*Atp6v0d2*	5′-GTGAGACCTTGGAA GACCTGAA-3′	5′-GAG​AAA​TGT​GCT​CAG​GGG​CT-3′
*Mmp9*	5′-CGT​GTC​TGG​AGA​TTC​GAC​TTG​A-3′	5′-TTG​GAA​ACT​CAC​ACG​CCA​GA-3′
*Actb*	5′-CAC​TGT​GCC​CAT​CTA​CGA-3′	5′-TGA​TGT​CAC​GCA​CGA​TTT-3′
*Nrf2*	5′-TCT​CCT​CGC​TGG​AAA​AAG​AA-3′	5′-AAT​GTG​CTG​GCT​GTG​CTT​TA-3′
*Keap1*	5′-TGC​CCC​TGT​GGT​CAA​AGT​G-3′	5*′*-GGT​TCG​GTT​ACC​GTC​CTG​C-3′
*CAT*	5′-CTC​GCA​GAG​ACC​TGA​TGT​CC-3′	5′-GAC​CCC​GCG​GTC​ATG​ATA​TT-3′
*GSR*	5*′*-GAC​ACC​TCT​TCC​TTC​GAC​TAC​C-3′	5*′*-CAC​ATC​CAA​CAT​TCA​CGC​AAG-3′
*HMOX-1*	5′-GAG​CAG​AAC​CAG​CCT​GAA​CT-3′	5*′*-AAA​TCC​TGG​GGC​ATG​CTG​TC-3*′*
*Nox2*	5′-ACC​GCC​ATC​CAC​ACA​ATT​G-3′	5′-CCG​ATG​TCA​GAG​AGA​GCT​ATT​GAA-3′
*Nqo1*	5′-TTC​TCT​GGC​CGA​TTC​AGA​G-3′	5′-GGC​TGC​TTG​GAG​CAA​AAT​AG-3′
*Trx1*	5′-TGC​TAC​GTG​GTG​TGG​ACC​TTG​C-3′	5′-ACC​GGA​GAA​CTC​CCC​CAC​CT-3′
*Sirt1*	5′-CCG​TTT​ATT​TTC​GCC​GTC​CGC​CAT​C-3′	5′-CGA​ACC​AAA​CTC​ACC​AAT​CTG​TGG​C-3′
*Sirt3*	5*′*-GCT​GCT​TCT​GCG​GCT​CTA​TAC-3′	5*′*-GAA​GGA​CCT​TCG​ACA​GAC​CGT-3′
*Sod1*	5*′*-AAC​CAG​TTG​TGT​TGT​CAG​GAC-3*′*	5*′*-CCA​CCA​TGT​TTC​TTA​GAG​TGA​GG-3*′*
*Hprt1*	5′-CAG​TCC​CAG​CGT​CGT​GAT​TA-3′	5′-TGG​CCT​CCC​ATC​TCC​TTC​AT-3′

qRT-PCR, quantitative real-time PCR; *Acp5*, acid phosphatase 5, tartrate-resistant; *Atp6v0d2*, vacuolar H+ transporting ATPase, V0 subunit D2; *c-fos*, proto-oncogene C-Fos; *Ctsk*, cathepsin K; *Mmp9,* matrix metallopeptidase 9; *Nfatc1*, nuclear factor of activated T cells 1; *Actb*, actin beta; *Nrf2,* nuclear factor E2-related factor 2; *Keap1,* kelch-like ECH-associated protein 1; *CAT,* catalase; *GSR,* glutathione reductase; *HMOX-1,* heme oxygenase-1; *Nox2,* NAD(P)H oxidase 2; *Nqo1,* NAD(P)H dehydrogenase quinone 1; *Trx1,* thioredoxin 1; *Sirt1,* sirtuin 1; *Sirt3,* sirtuin 3; *Sod1,* superoxide dismutase 1; *Hprt1,* hypoxanthine phosphoribosyltransferase 1.

### Luciferase Activity Assay

RAW264.7 cell lines transfected with NF-κB, NFATc1, and ARE luciferase reporter genes were used following the previous methods (Qiu et al., 2021), and these cell lines were cultured by D-MEM containing 10% FBS, 1% PS. The next day, after being treated with CAG (2.5, 5, 10 μM) for 60 min, the cells were stimulated by RANKL for stated durations, which were 6 h (NF-κB), 24 h (NFATc1), and 48 h (Nrf2/ARE). After that, these cells were lysed and centrifuged, and the supernatants of 50 μL were loaded to each well of a 96-well microplate. The readings of luciferase activity were performed by an automated multi-purpose plate reader.

### Immunoblot Assay

For short-term effect detection of CAG on protein expression, 1.5×10^5^ BMMs per well on 6-well plates were co-stimulated by RANKL and CAG (10 μM) for 10, 20, 30, or 60 min. For long-term effect detection of CAG in BMMs, cells were seeded with 1×10^5^ cells per well, and the duration of RANKL and CAG (10 μM) were 1, 3, or 5 days, respectively. Afterward, a radio-immunoprecipitation assay buffer was applied to extract proteins from the harvested cells. These proteins were then electrophoresed in a 10% gel and transferred to a nitrocellulose membrane. After being blocked by 5% non-fat milk, the membrane was incubated with primary antibody at 4°C and secondary antibody at room temperature (RT) for 10 and 1 h, respectively. After that, the membrane soaked by an enhanced reagent (PerkinElmer, Waltham, MA) was applied to the Image-quant LAS system (GE Healthcare, Silverwater, Australia) for development. Relative protein expression was normalized to that of the housekeeping protein.

### Calcium Oscillation Assay

BMMs were cultured on non-adjacent wells of a 48-well plate at a density of 1.5×10^4^ cells/well. The next day, cells were first treated with CAG (10 μM) for 1 h and then stimulated by RANKL for 24 h without CAG removal. An assay buffer composed of HANKS balanced salt solution, 1% FBS, and 250 mM probenecid was utilized for cell rinsing. Under dark and RT conditions, a staining buffer prepared by mixing 4 μM Fluo4-AM with 20% (w/v) pluronic-F127/DMSO solution was used for cell incubation of 45 min. Cells were triple rinsed and then incubated for 20 min with the assay buffer. After another assay buffer replacement, the plate was mounted on a fluorescence microscope with a wavelength set to 488 nm for a delayed shot of 2 min-long, with an interval of 2 s. Flicker cells capable of displaying maximum brightness more than 2 times were viewed as calcium oscillation cells, and the signal intensity was recorded as the difference between the maximum and minimum intensity of brightness.

### OVX-Induced PMO in Mice

Under the approval of the Animal Ethics Committee of Guangzhou University of Chinese Medicine (GZUCM) (Ethics NO. 20190328002), eighteen 10-week-old female C57BL/6J mice were obtained from the Animal Center of GZUCM and randomly divided into three groups of the same number (n = 6), namely the sham group, the OVX group, and the CAG-treated OVX group. Mice in the latter two OVX groups received bilateral ovariectomy, while mice in the sham group underwent ovarian exposure operation. After 1 week of recuperation, mice in the CAG-treated group received intraperitoneal injection of CAG (10 mg/kg). Meanwhile, an equal volume of PBS was injected into the abdominal cavity of mice in the other two groups. Both CAG and PBS were used once every 2 days for 7 weeks. After that, all mice were euthanized for femurs collection.

### Micro-CT Assay

The right femur of these mice was carefully stripped of the soft tissue on its surface and then immersed in 4% paraformaldehyde (PFA) for 48 h. Later, a Skyscan 1176 micro-CT scanner (Bruker, Kontich, Belgium) was appointed to scan the middle and lower segments of these femurs. The volume 1.5 mm above the distal epiphyseal line and 1 mm in height was selected as the region of interest (ROI). The scanning parameters were the same as those used previously (Liu et al., 2019). The trabecular bone parameters of the ROI, including bone volume/tissue volume (BV/TV), trabecular number (Tb. N), trabecular thickness (Tb. Th), and connectivity density (Conn.Dn), was calculated through the included CTAn program. Representative 2D and 3D images of the trabecular bone were synthesized using the included DataViewer and CTvol program, respectively.

### Statistical Analysis

Trials in this study included at least three repetitions, and the data obtained were presented as mean and standard deviation (SD). The comparison between two groups at different time points and the comparison between different groups were carried out by two-way ANOVA and one-way ANOVA, respectively. All statistics were performed on GraphPad Prism 9.0 software, and a *p*-value less than 0.05 was considered statistically significant.

## Results

### CAG Dose- and Time-Dependently Inhibits RANKL-Induced Osteoclastogenesis

**FIGURE 1:**
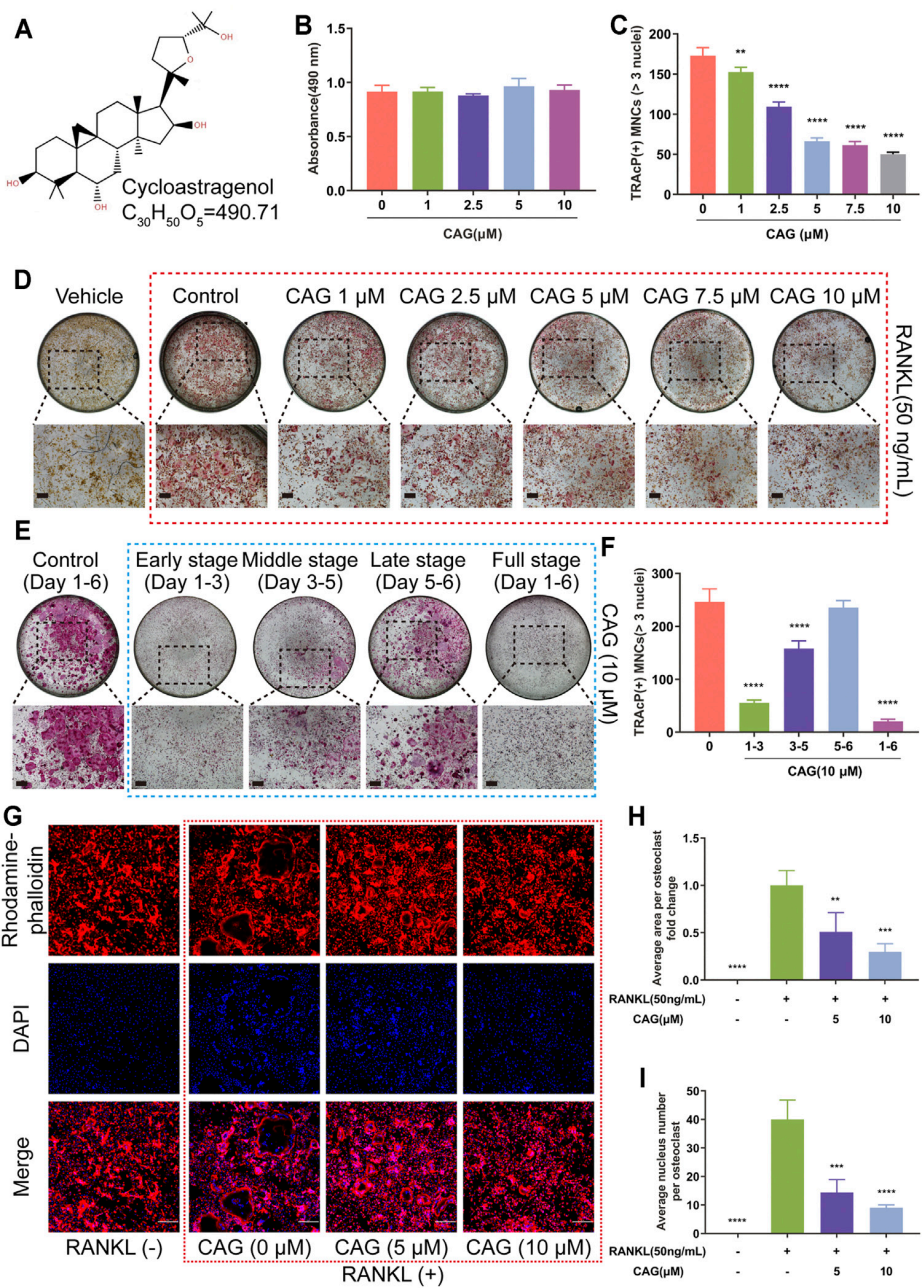
CAG inhibits osteoclastogenesis and osteoclastic F-actin belt formation triggered by RANKL in BMMs. **(A)** The structural formula and molecular formula of CAG. **(B)** MTS test demonstrated that the proliferation of BMMs was not affected by different concentrations of CAG for up to 48 h. **(C)** Quantitative analysis exhibited that the rise of CAG concentration resulted in the decline of osteoclast count. **(D)** Typical TRAcP staining images of osteoclasts formed by BMMs after being treated with 1, 2.5, 5, 7.5, and 10 µM of CAG for 6 days. **(E)** Quantitative analysis indicated that CAG administration on days 1–3 and 3–5 reduced the osteoclast count on day 6. **(F)** Typical TRAcP staining images of osteoclasts formed by CAG-treated BMMs on days 1–3, days 3–5, days 5–6, and days 1–6. **(G)** Typical immunostaining images of F-actin rings (red belts) and nuclei (blue dots) formed by BMMs after treating them with 5 and 10 µM of CAG for 6 days. **(H–I)** Quantitative analysis unveiled that the gradual decrease in the area by F-actin and the number of nuclei were correlated with the increase in CAG concentration. The largest osteoclasts in each group were selected to count nuclear numbers. n = 32 (CAG 0 µM), 18 (CAG 5 µM), and 19 (CAG 10 µM) (n = osteoclasts numbers). Data of all histograms are displayed as mean (SD). n = 3. **p < 0.01, ***p < 0.001. ****p < 0.0001. Scale bar = 200 µM. BMMs: bone marrow monocytes; CAG: cycloastragenol; DAPI: 4′,6-diamidino-2-phenylindole; MNCs: multinucleated cells; MTS: cell proliferation assay; RANKL: receptor activator of nuclear factor κB ligand; TRAcP: tartrate-resistant acid phosphatase.

The formula and structure of CAG are displayed in Figure 1A. In our previous drug screening, CAG showed no promoting effect on angiogenesis and osteoblastogenesis *in vitro* (Supplementary Figures S1,S2). To determine the effect of CAG on osteoclastogenesis, different concentration of CAG were used to treat BMMs. BMMs exhibited no apparent cytotoxicity in the subsequent MTS test after being treated with CAG of 1–10 μM for 2 days (Figure 1B and Supplementary Figure S3). Interestingly, the count of MNCs with more than three nuclei stained positively for TRAcP revealed that the increase of CAG doses was correlated with the depletion in the number of RANKL-induced osteoclasts (Figures 1C,D). Furthermore, TRAcP staining revealed that the inhibitory effect of CAG was time-dependent, which was strongest in the early stage (Day 1–3), followed by the middle stage (Day 3–5), but was unremarkably in the late stage (Day 5–6) (Figures 1E,F). Immunofluorescence (IF) staining also verified the dose-dependent inhibition of CAG on RANKL-induced osteoclastogenesis, manifested by the impaired area by F-actin belts and the decreased nuclei number in CAG-treated cells (Figures 1G–I).

### CAG Constrains RANKL-Induced Bone Resorption and Osteoclast-Specific Gene Expression

**FIGURE 2:**
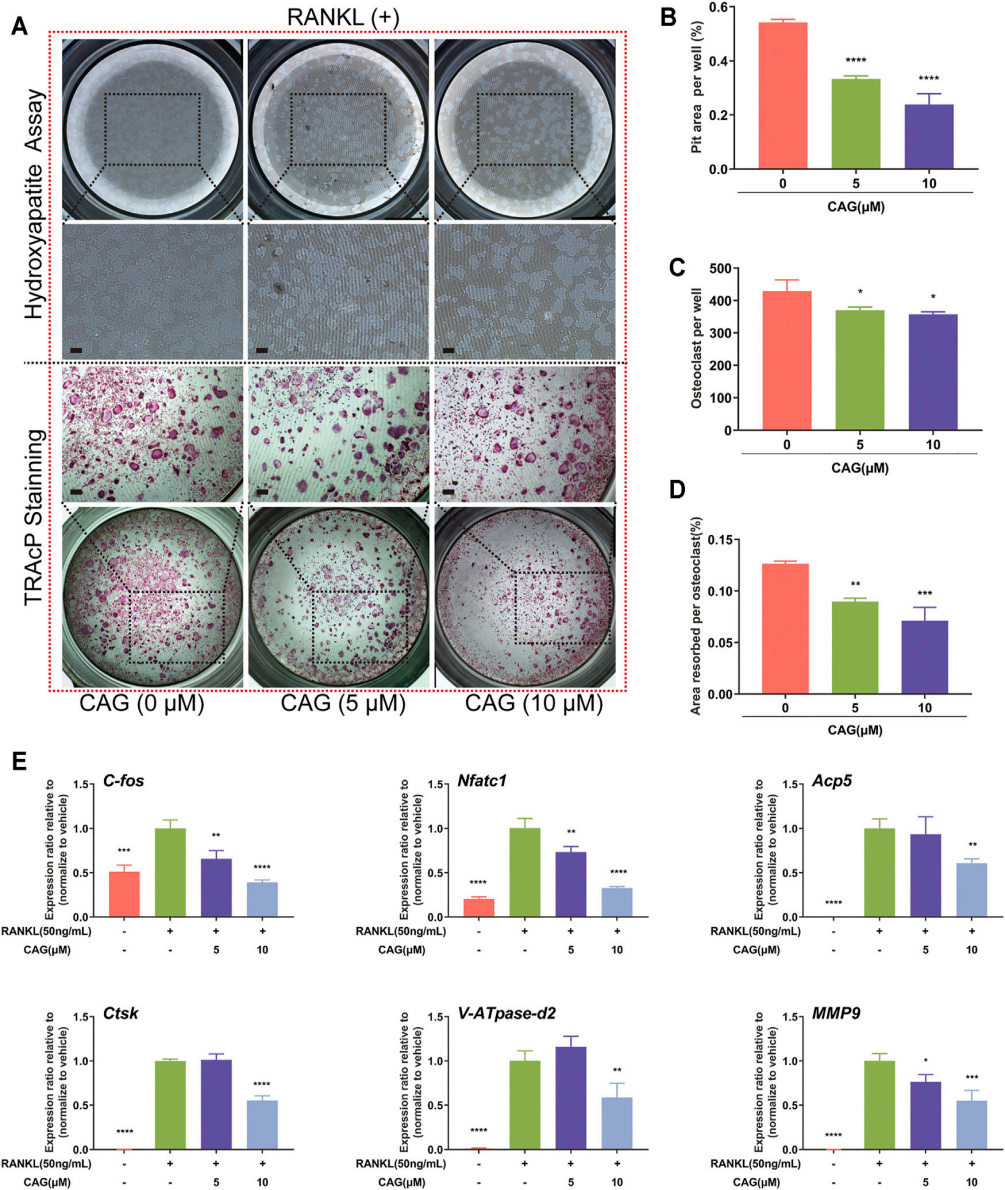
CAG blocks bone resorption and osteoclast-specific gene expression in RANKL stimulated BMMs. **(A)** Typical images of resorption pits and TRAcP staining in BMMs treated with 5,10 µM of CAG for 6 days **(B–D)** The quantitative comparison showed that the pits area per well, the osteoclasts number, and the ratio of the two (i.e., the pits area of a single osteoclast) decreased stepwise in BMMs treated with 5 and 10 µM CAG. **(E)** Quantitative comparison of real-time qPCR showed that osteoclast-specific gene expression, including *c-Fos*, *Acp5*, *Nfatc1*, *Atp6v0d2*, *Ctsk*, and *Mmp9,* declined stepwise in BMMs treated with 5 and 10 µM CAG. Data of all histograms are displayed as mean (SD). n = 3. *p < 0.05, **p < 0.01, ***p < 0.001. ****p < 0.0001. Scale bar = 200 µM. RANKL: receptor activator of nuclear factor κB ligand; CAG: cycloastragenol; TRAcP: tartrate-resistant acid phosphatase; *c-Fos*: proto-oncogene c-Fos; *Acp5*: acid phosphatase 5; *Nfatc1*: nuclear factor of activated T cells 1; *Atp6v0d2*: vacuolar H^+^ transporting ATPase V0 subunit D2; *Ctsk*: cathepsin K; *Mmp9*: matrix metallopeptidase 9.

BMMs were stimulated to form osteoclasts on the hydroxyapatite surface. Consistent with the above findings, TRAcP staining showed that the increase of CAG doses was associated with the gradual decrease of osteoclast number. Furthermore, a dose-dependent effect of CAG on osteoclast function was discovered by comparing the area of small pits of eroding hydroxyapatite by osteoclasts (Figures 2A–D). In terms of cellular mechanism, the influence of CAG on osteoclast-specific genes was assessed by real-time qPCR assay. As expected, the relative expression level of *c-Fos*, *Acp5*, *Nfatc1*, *Atp6v0d2*, *Ctsk*, and *Mmp9* in CAG-treated BMMs was restricted, and the level of restriction was correlated with the doses of CAG (Figure 2E).

### CAG Impedes NF-κB Pathwa*y* While Enhancing Nrf2/Keap1/ARE Pathway

**FIGURE 3:**
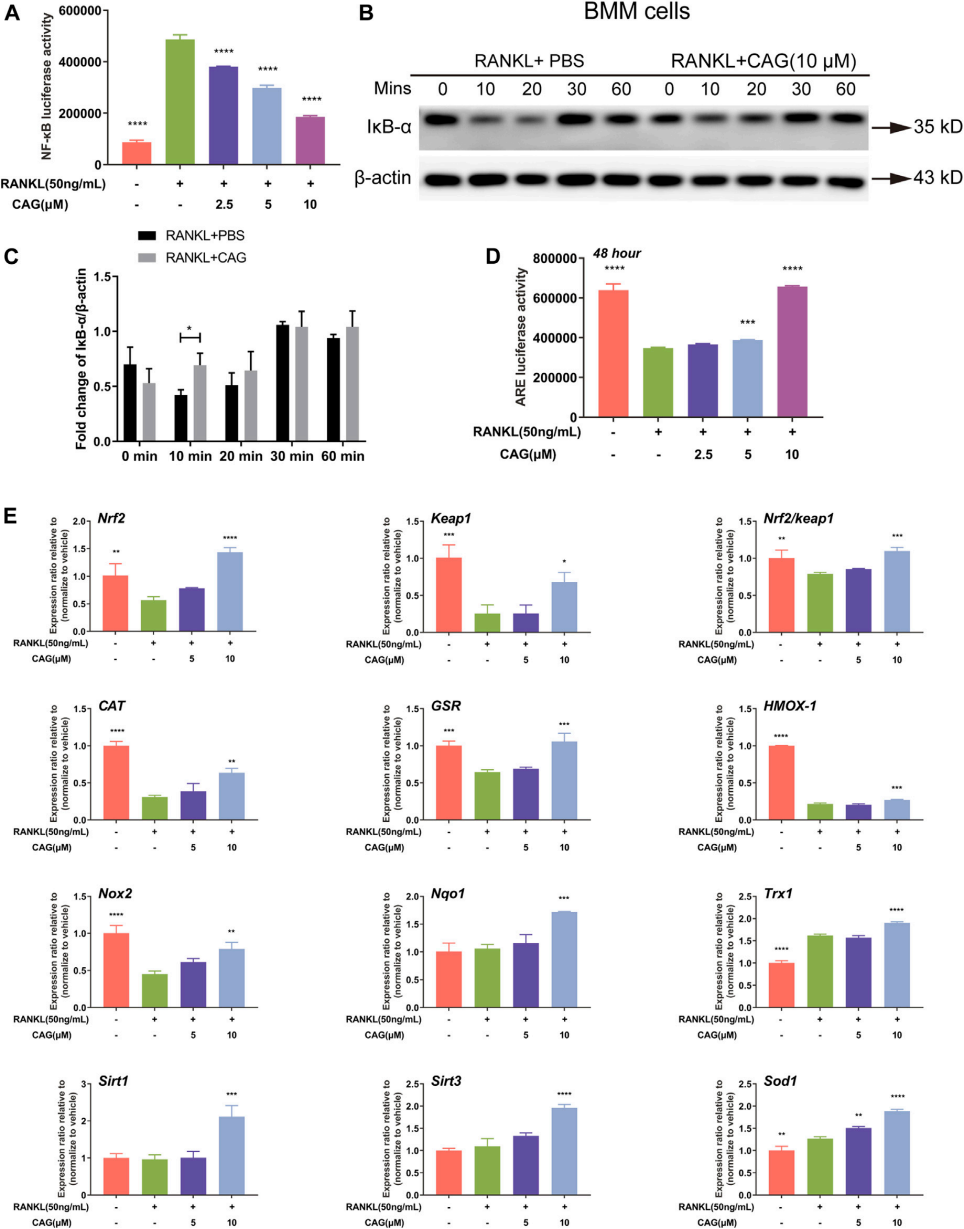
CAG inhibits the NF-κB pathway and promotes Nrf2/Keap1/ARE pathway. **(A)** Luciferase reporter gene assay in RAW264.7 cells presented that the activity of NF-κB triggered by RANKL decreased after treated with 2.5, 5, and 10 µM CAG. **(B)** Typical western blot images of protein expression in BMMs treated with 10 µM CAG for 10, 20, 30, and 60 min. **(C)** Quantitation revealed that the relative expression of IκB-α protein significantly increased after BMMs were treated with CAG at 10 min. **(D)** Luciferase reporter gene assay in RAW264.7 cells proved that CAG administration dose-dependently increased the activity of ARE suppressed by RANKL. **(E)** Real-time qPCR assay demonstrated that CAG treatment of 5 and 10 µM led the expression of genes in the Nrf2/Keap1 pathway and the Nrf2/Keap1 ratio to rise dose-dependently. Data of all histograms are displayed as mean (SD). n = 3. *p < 0.05, **p < 0.01, ***p < 0.001. ****p < 0.0001. NF-κB: nuclear factor-κB; RANKL: receptor activator of nuclear factor κB ligand; CAG: cycloastragenol; BMMs: bone marrow monocytes; PBS: phosphate-buffered saline; IκB-α: inhibitor kappa B-alpha; ARE, anti-oxidant response element; *Nrf2,* nuclear factor E2-related factor 2; *Keap1,* kelch-like ECH-associated protein 1; *CAT,* catalase; *GSR,* glutathione reductase; *HMOX-1,* heme oxygenase-1; *Nox2,* NAD(P)H oxidase 2; *Nqo1,* NAD(P)H dehydrogenase quinone 1; *Trx1,* thioredoxin 1; *Sirt1,* Sirtuin 1; *Sirt3,* Sirtuin 3; *Sod1,* superoxide dismutase 1.

NF-κB activation by RANKL was found in RAW264.7 cells by luciferase reporter assay. Interestingly, CAG was shown to have a dose-dependent inhibition on NF-κB activity in these cells (Figure 3A). The proteolysis of IκB–α serves as a landmark of the RANKL-stimulated NF-κB pathway. Western blot indicated that RANKL when acting as a short-term stimulus, caused the reduction of IκB–α protein expression level in BMMs, which was reversed by CAG, and this effect was most significant at 10 min (Figures 3B,C). Further, luciferase reporter assay showed that 48 h of RANKL stimulation led to impairment of ARE activity in RAW264.7 cells (Figure 3D). Nrf2/Keap1/ARE axis plays a pivotal role in promoting ROS-eliminating enzymes. Through real-time qPCR assay, CAG-treated BMMs were found to have higher mRNA expression of Nrf2 and other antioxidant enzymes, as well as a higher Nrf2/Keap1 ratio than CAG-untreated cells, and these effects were dose-dependent (Figure 3E).

### CAG Restrains Calcium-NFATc1 Pathway

**FIGURE 4:**
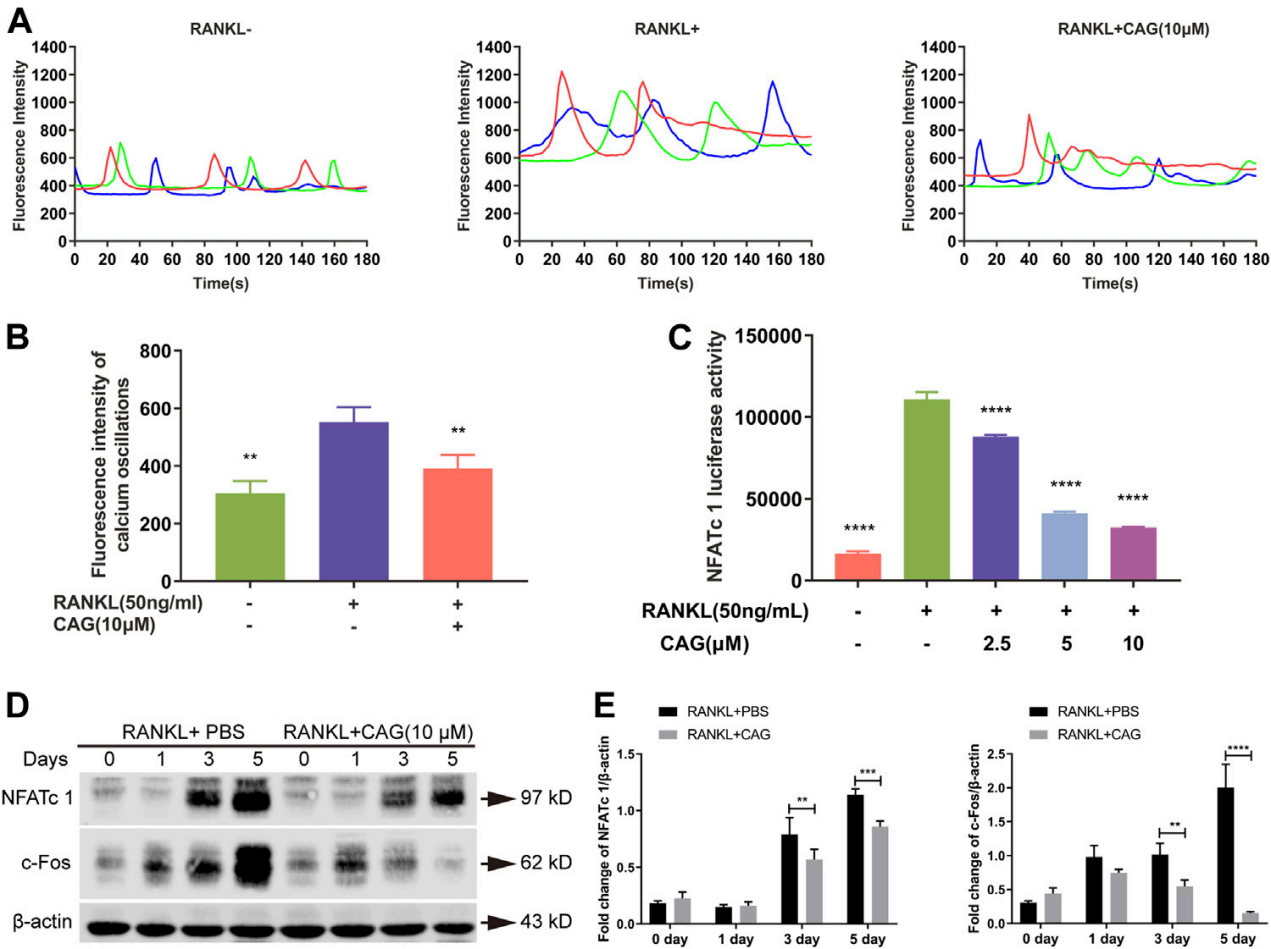
CAG restrains RANKL-induced Ca^2+^/NFATc1 pathway. **(A)** Fluorescence intensity curves of BMMs loaded with Fluo4-AM calcium probe after being treated with 10 µM CAG for 48 h. **(B)** Quantitation of fluorescence intensity disclosed that CAG administration caused a lessening in the intensity of calcium oscillations in BMMs stimulated by RANKL. **(C)** Luciferase reporter assay in RAW264.7 cells exhibited that 2.5, 5, and 10 µM CAG led to a reduction in NFATc1 activity. **(D)** Typical western blot images of protein expression in BMMs after being treated with CAG for 1, 3, and 5 days. **(E)** Quantitative comparison indicated that 3 and 5 days of CAG treatment caused a significant decrease in the protein expression of c-Fos and NFATc1 in BMMs. Data of all histograms are displayed as mean (SD). n = 3. *p < 0.05, **p < 0.01, ***p < 0.001. ****p < 0.0001. RANKL: receptor activator of nuclear factor κB ligand; CAG: cycloastragenol; NFATc1: nuclear factor of activated T cells 1; BMMs: bone marrow monocytes; PBS: phosphate-buffered saline; c-Fos: proto-oncogene C-Fos.

Calcium oscillation by RANKL stimulation was monitored in BMMs loaded with a Fluo4-AM fluorescent probe. Compared with the untreated group, CAG-treated cells were observed to have a weaken calcium oscillation (Figures 4A,B). The activation of NFATc1 provoked by RANKL was decreased by CAG (Figure 4C). C-Fos plays a crucial role in the transcriptional activation of NFATc1 in RANKL-induced signaling. As shown in the western blot assay, RANKL stimulation led to elevated expression of c-Fos and NFATc1, while the application of CAG reversed these effects in BMMs (Figures 4D,E).

### CAG Prevents Bone Loss in OVX Mice

**FIGURE 5:**
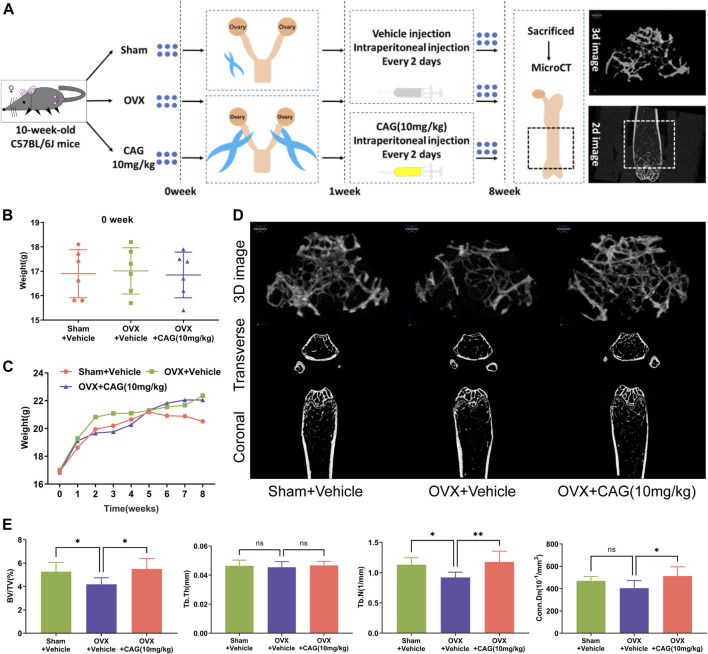
CAG alleviates MPO-related bone loss in mice. **(A)** Flow chart of PMO model induced by OVX in female C57BL/6J mice. **(B)** The initial body weight of mice had a consistent baseline in different groups. **(C)** Weekly body weight changes of mice in different groups were recorded. **(D)** Typical 2D and 3D images of the distal femur of MPO mice were reconstructed by MicroCT scanning after 7 weeks of exposure to CAG (10 mg/kg). **(E)** Quantitative analysis exhibited that CAG management markable improved the bone parameters of femoral ROI in MPO mice, including BV/TV, Tb. N, and Conn. Dn. Data of all histograms are displayed as mean (SD). n = 3. *p < 0.05, **p < 0.01. OVX, ovariectomize; CAG, cycloastragenol; 2D, two-dimension; 3D, three-dimension; BV/TV, bone volume/tissue volume; Tb. N, trabecular number; Tb. Th, trabecular thickness; Conn. Dn, connectivity density.

The effect of CAG on bone loss *in vivo* was carried out according to the protocol in Figure 5A. No significant difference was found in the initial body weight between the three groups (Figure 5B), and the weekly body weight changes of each group were recorded (Figure 5C). Data from Micro-CT scanning were utilized for the measurement of the bone parameter. Representative 2D and 3D images of the ROI of the femur exhibited bone deterioration in OVX mice, while less bone damage was seen in mice exposed to CAG (10 mg/kg) (Figure 5D). Compared with the sham group, reduction of BV/TV and Tb. N was determined in the OVX group, but no significant difference existed between these two groups in terms of Tb. Th and Conn. Dn. In contrast, augment of BV/TV, Tb. N, and Conn. Dn was noted in the CAG-treated group when compared to the OVX group, but no significant difference in Tb. Th between these two groups (Figure 5E).

## Discussion

Bone remodeling maintains a highly regulated balance between osteoclastic degradation and osteoblastic deposition in healthy individuals (Hadjidakis and Androulakis, 2006). This balance, however, is disrupted in PMO and other osteolytic diseases, favoring the osteoclast activity (Crotti et al., 2015). Osteoclast precursors come from the monocyte/macrophage system, proliferate under initial activation of the M-CSF/c-FMS pathway, and become TRAcP positive multinucleated cells under further activation of the RANKL/RANK pathway. Mature osteoclasts anchor to the bone surface through their structure mainly composed of F-actin rings (Lakkakorpi et al., 1989). With the participation of V-ATPase pump, protons and various proteinases, such as TRAcP (*Acp5*), CTSK (*Ctsk*), and MMP9 (*Mmp9*) (Delaissé et al., 2003), were released to degrade bone matrix in the sealing zone enclosed by the ruffled border (Blair et al., 1989; Väänänen et al., 1990). In our study, TRAcP staining, F-actin belt staining, and pit-resorbing assay respectively confirmed that CAG treatment restrained osteoclast formation, F-actin belt formation and bone resorption induced by RANKL. RT-qPCR assay then confirmed the suppression of CAG treatment on downstream genes specific to osteoclast-forming and bone-resorbing, which explains the biologic changes in osteoclasts, and further indicates the potential inhibitory effects on the upstream of RANKL-induced signaling.

ROS, generally considered as a second messenger, is a signpost in the formation of osteoclasts activated by RANKL (Ha et al., 2004). Exogenous antioxidants, such as N-acetyl-l-cysteine or glutathione, have been found to inhibit osteoclastogenesis, F-actin ring formation, and function of osteoclasts induced by RANKL by eliminating ROS (Ha et al., 2004). Nrf2/Keap1/ARE axis is the principal endogenous pathway that mediates ROS clearance (Hyeon et al., 2013; Kanzaki et al., 2013). Under the stimulation of RANKL, the cleaved Keap1 loses association with Nrf2, causing the latter to escape and enter the nuclear, where it binds to the ARE promoter region to boost the expression of antioxidant enzymes (Kanzaki et al., 2013). Overexpression of Keap1 or knockdown of Nrf2 were associated with increased RANKL-induced ROS production, osteoclastogenesis, and bone resorption via attenuating cytoprotective enzymes like NQO1 and HO-1 (Kanzaki et al., 2013). Through luciferase reporter gene assay, CAG treatment was found to augment the transcriptional activity of ARE blocked by RANKL. Consistent with this, CAG addition also up-regulated Nrf2 expression, Nrf2/Keap1 ratio, and the induction of antioxidant enzymes as seen in RT-qPCR. Based on these pieces of evidence, the regulation of the RANKL-induced Nrf2/Keap1/ARE pathway may play a potential role in CAG’s inhibitory effect on RANKL-induced osteoclastogenesis.

NF-κB of mammal species are dimers formed by the multiple combinations of five protein subtypes, which are p50 (NFKB1), p52 (NFKB2), p65 (RelA), c-Rel (Rel), and RelB, all sharing a Rel homologous domain. The vital role of NF-κB in osteoclast activation has been confirmed by the fact that double knockout of p50/p52 in mice led to an osteopetrosis phenotype due to osteoclast defects (Franzoso et al., 1997; Iotsova et al., 1997). Under RANKL stimulation, the classical NF-κB pathway was activated, and major events include IκB–α phosphorylation and degradation, escape of NF-κB from IκB–α/NF-κB complex into nucleus, and the attachment of NF-κB to the promoter of downstream genes. Not surprisingly, inhibition of the classical pathway was found to decrease osteoclast-forming and bone-resorbing capacity (Jimi et al., 2004; Soysa et al., 2009). In contrast, constitutive activation of the classical pathway resulted in aggravated bone loss in mice (Otero et al., 2012). For the evaluation of CAG on the activity of the NF-κB pathway, luciferase assay and western blot assay were separately performed. Dramatically, the results exhibited that CAG caused a reduction in NF-κB activity and IκB–α degradation. Based on the above, the inhibitory effect of CAG on the NF-κB pathway plays a part in RANKL-induced signaling.

NFATc1 (NFAT2), a subtype of NFAT transcription factor, serves as a master transcriptional factor of osteoclast differentiation and function induced by RANKL (Takayanagi et al., 2002). Importantly, stable calcium oscillations, induced by RANKL, promote NFATc1 nuclear translocation (and auto-amplification) and thus osteoclast differentiation through a calcineurin-dependent pathway. In addition, it was also demonstrated that forced expression of NFATc1 could rescue osteoclast differentiation in cells deprived of c-Fos, which the latter is also a key transcription factor of osteoclastogenesis (Grigoriadis et al., 1994; Takayanagi et al., 2002). Besides, the calmodulin/cAMP-responsive element-binding protein (CREB) pathway activated by calcium ossification was found to regulate the expression of c-Fos, which participate in the promoter binding of osteoclast-specific genes cooperatively with NFATc1 (Sato et al., 2006). As seen in our calcium oscillation assay and western blot, RANKL induced calcium mobilization and protein expression of NFATc1 and c-Fos in osteoclast precursor cells, while CAG intervention revised these phenomena. Correspondingly, by luciferase reporter assays, CAG was also found to exhibit a reduced NFATc1 activity in osteoclast precursor cells.

**FIGURE 6:**
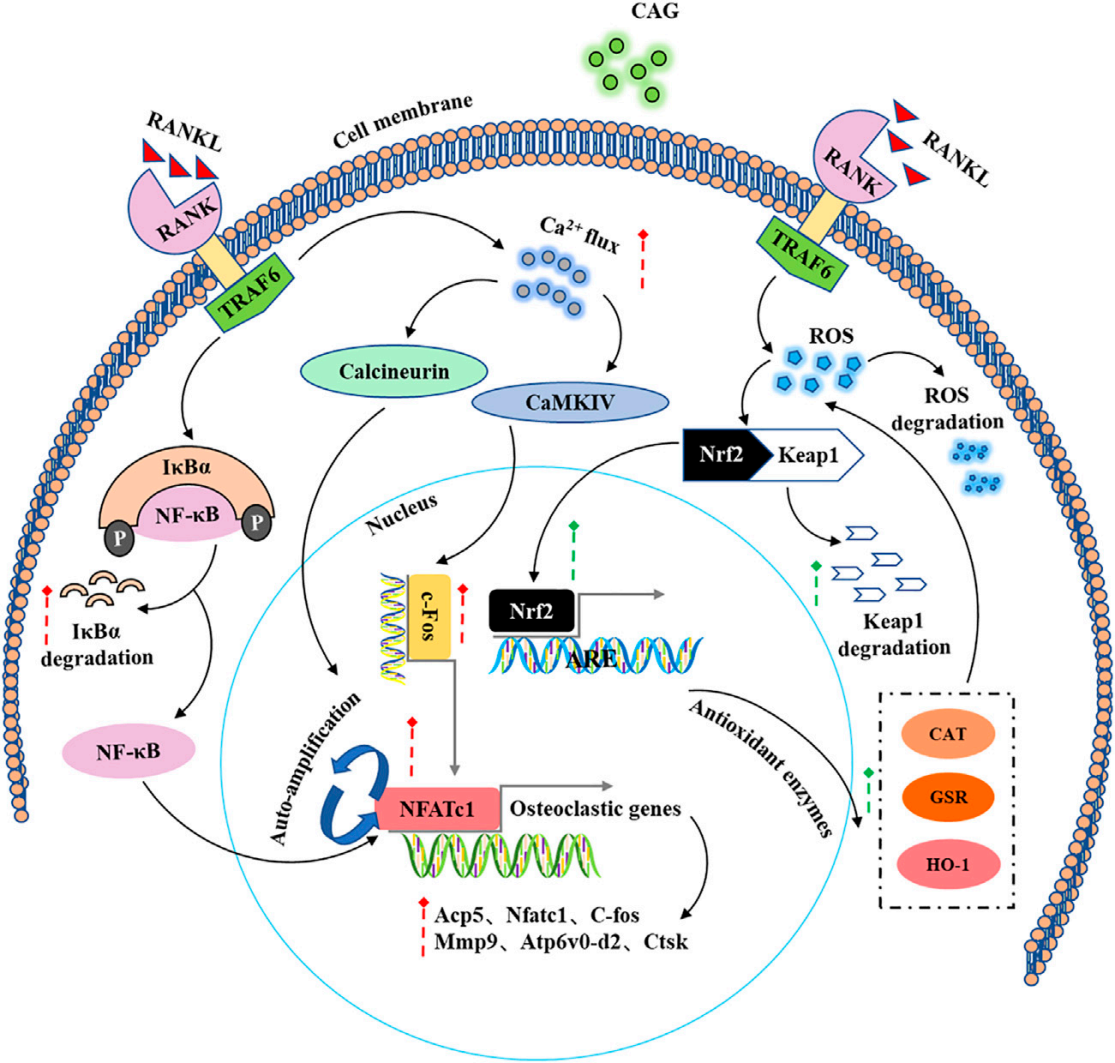
A schematic diagram of the regulatory effect of CAG in the intracellular signaling pathways induced by RANKL.

Based on the effects mentioned above of CAG on osteoclast differentiation and function and its effects on RANKL-induced intracellular signaling pathways (Figure 6), a PMO model was carried out in mice to determine the protective effect of CAG on osteoporosis. Consistently, the data from micro-CT scanning demonstrated a reduced bone loss in CAG-treated mice, which was reflected in the increase of bone parameters, including BV/TV, Tb. N, and Conn. Dn. We found that OVX led to total body weight gain in mice, which is usually attributed to the fluctuation of the hormonal milieu (Davis et al., 2012). In contrast, the weight change was relatively low in CAG-intervened mice, the mechanism of which needs to be further studied.

## Conclusions

Taken together, our data showed that CAG prevents RANKL-induced osteoclast-forming and bone-resorbing *in vitro* via enhancing ROS scavenging (Nrf2/Keap1/ARE) pathway, and suppressing NF-κB and calcium pathway, and thus leads to the downregulation of crucial transcript factors, including NFATc1 and c-Fos. Based on our preclinical experimental results, CAG may serve as an alternative drug for the potential treatment of osteoclast-related bone diseases such as PMO.

## Data Availability

The original contributions presented in the study are included in the article/Supplementary Material, further inquiries can be directed to the corresponding authors.
