# Supplementary material for: Cycloastragenol Attenuates Osteoclastogenesis and Bone Loss by Targeting RANKL-Induced Nrf2/Keap1/ARE, NF-κB, Calcium, and NFATc1 Pathways
Source: Front Pharmacol. 2022 Jan 20;12:810322. doi: 10.3389/fphar.2021.810322 (PMC8812338; doi:10.3389/fphar.2021.810322)
Supplement: Supplementary file 1 [file Table2.DOCX]

C:\Users\王刚\Desktop\7.细胞试验\wb\20181212 #4 #7 bmm 长效 续20181205\4





#4 M1 15S 2 BACTIN 20181212





cfos #4 M1 15S 20181212





integrin #4 M1 5S 2 20181212





nfatc1 #4 M1 5S 20181212

C:\Users\王刚\Desktop\7.细胞试验\wb\20181212 #4 #7 bmm 长效 续20181205\4





**#4 M2 15S D2 BACTIN 20181212**





**cfos #4 M2 10S 20181212**





**integrin #4 M2 10S 20181212**





**nfatc1 #4 M2 5S 20181212**

**C:\Users\王刚\Desktop\7.细胞试验\wb\20181231 #4 #5 #7 bmm 长效\4**





**#4 5 7 M1 2 INTEGRIN 150S 20190101**





**#4 M1 CFOS 15S 20181231**





**#4 M1 D2 beta actin 15S 20181231**





**#4 M1 INTEGRIN 10S 20181231**





**#4 M1 NFATC1 2S 20181231**





**#4 M2 CFOS 15S 20181231**





**#4 M2 D2 beta actin 20S 20190102**





**#4 M2 INTEGRIN 10S 20181231**





**#4 M2 NFATC1 2S 20181231**

**C:\Users\王刚\Desktop\7.细胞试验\wb\20190105 #4 #5 #7 bmm 长效 续20181231**





**#4 M3 D2 15S 20190105**





**#4 M3 CFOS 5S 20190105**





**#4 M3 INTEGRIN 15S 20190105**





**#4 M3 NFATC1 2S 20190105**





**#4 M4 CTSK 10S 20190105**





**#4 M4 CFOS 10S 20190105**





**#4 M4 INTEGRIN 15S 20190105**





**#4 M4 NFATC1 2S 20190105**

**C:\Users\王刚\Desktop\7.细胞试验\wb\20190128 #4 #7 bmm 长效 续20181231 20190105\4 bmm 20190128 long effect**





**#4 mo1 bactin 20s 20190128_1508**





**#4 mo1 cfos 30s 20190128_1508**





**#4 mo1 integrin 50s 20190128_1508**





**#4 mo1 nfatc1 5s 20190128_1508**





**#4 mo2 cfos 30s 20190128_1508**





**#4 mo2 d2 bactin 30s 20190128_1508**





**#4 mo2 integrin 50s 20190128_1508**





**#4 mo2 nfatc1 5s 20190128_1508**





**#4 mo3 bactin 20s 20190128_1508**





**#4 mo3 cfos 30s 20190128_1508**





**#4 mo3 integrin 50s 20190128_1508**





**#4 mo3 nfatc1 10s 20190128_1508**





**#4 mo4 ctsk 30s 20190128_1508**





**#4 mo4 cfos 30s 20190128_1508**





**#4 mo4 integrin 50s 20190128_1508**





**#4 mo4 nfatc1 10s 20190128_1508**

**C:\Users\王刚\Desktop\7.细胞试验\wb\20190106 #4 #5 #7 raw 长效\4 RAW CELL**





**#4 M1 bactin 3S 20190109 RAW CELL**





**#4 M1 CFOS 50S 20190106 RAW CELL**





**#4 M1 INTEGRIN 30S 20190106 RAW CELL**





**#4 M1 NFATC1 40S 20190106 RAW CELL**





**#4 M2 bactin 3S 20190109 RAW CELL**





**#4 M2 CFOS 50S 20190106 RAW CELL**





**#4 M2 INTEGRIN 30S 20190106 RAW CELL**





**#4 M2 NFATC1 40S 20190106 RAW CELL**





**#4 M3 bactin 3S 20190109 RAW CELL**





**#4 M3 CFOS 50S 20190106 RAW CELL**





**#4 M3 INTEGRIN 30S 20190106 RAW CELL**



 **#4 M3 NFATC1 20S 20190106 RAW CELL**

**短效short effect**

**C:\Users\王刚\Desktop\7.细胞试验\wb\20190613 #4 bmm 2019210 短效**





**C:\Users\王刚\Desktop\7.细胞试验\wb\20190613 #4 bmm 2019210 短效\IKBA 1 120S 2**





**C:\Users\王刚\Desktop\7.细胞试验\wb\20190613 #4 bmm 2019210 短效\ACTIN 1 10S**





**C:\Users\王刚\Desktop\7.细胞试验\wb\20190613 #4 bmm 2019210 短效\IKBA 2 60S 2**





**C:\Users\王刚\Desktop\7.细胞试验\wb\20190613 #4 bmm 2019210 短效\ACTIN 2 10S**





**C:\Users\王刚\Desktop\7.细胞试验\wb\20190613 #4 bmm 2019210 短效\IKBA 3 120S 2**





**C:\Users\王刚\Desktop\7.细胞试验\wb\20190613 #4 bmm 2019210 短效\ACTIN 3 50S**





**C:\Users\王刚\Desktop\7.细胞试验\wb\20190613 #4 bmm 2019210 短效\IKBA 4 120S 2**





**C:\Users\王刚\Desktop\7.细胞试验\wb\20190613 #4 bmm 2019210 短效\ACTIN 4 30S**





**C:\Users\王刚\Desktop\7.细胞试验\wb\20181208 #4 #7 bmm 短效 IKBA #4 M2 20S 20181210**





**C:\Users\王刚\Desktop\7.细胞试验\wb\20181208 #4 #7 bmm 短效 bactin #4 M2 20S 20181210**





**#4 mo4 wushuxian ikba 60s 1 20190328_2108.gel**

**下列文件来源于 C:\Users\王刚\Desktop\7.细胞试验\wb\20190406 #4 #7 bmm p3 20190303 A1 是4号药物 A2 4是7号药物 短效b2 ikba 30s 1**





**b2 ikba 30s 2 #4 20190210 bmm**





**#B 4 BACTIN 60S 20190228 用的20190210 bmm #4样本 膜上顺序 0-60- 0+60+**
